# Dislocation following total knee arthroplasty: A report of six cases

**DOI:** 10.4103/0019-5413.69318

**Published:** 2010

**Authors:** Manuel Villanueva, Antonio Ríos-Luna, Javier Pereiro, Homid Fahandez-Saddi, Antonio Pérez-Caballer

**Affiliations:** Department of Orthopedics, Hospital General Universitario Gregorio Marañón, Madrid, Spain; 1 Department of Orthopedics, Hospital Virgen del Mar, Almería, Spain; 2 Department of Orthopedics, Hospital Clínico Universitario San Carlos, Madrid, Spain; 3 Department of Orthopedics, Hospital Fundación Alcorcón, Madrid, Spain; 4 Department of Orthopedics, Hospital Infanta Elena, Madrid, Spain

**Keywords:** Total knee arthroplasty, dislocation, instability

## Abstract

**Background:**

Dislocation following total knee arthroplasty (TKA) is the worst form of instability. The incidence is from 0.15 to 0.5%. We report six cases of TKA dislocation and analyze the patterns of dislocation and the factors related to each of them.

**Materials and Methods:**

Six patients with dislocation of knee following TKA are reported. The causes for the dislocations were an imbalance of the flexion gap (n=4), an inadequate selection of implants (n=1), malrotation of components (n=1) leading to incompetence of the extensor mechanism, or rupture of the medial collateral ligament (MCC). The patients presented complained of pain, giving way episodes, joint effusion and difficulty in climbing stairs. Five patients suffered posterior dislocation while one anterior dislocation. An urgent closed reduction of dislocation was performed under general anaesthesia in all patients. All patients were operated for residual instability by revision arthroplasty after a period of conservative treatment.

**Results:**

One patient had deep infection and knee was arthrodesed. Two patients have a minimal residual lag for active extension, including a patient with a previous patellectomy. Result was considered excellent or good in four cases and fair in one, without residual instability. Five out of six patients in our series had a cruciate retaining (CR) TKA designs: four were revised to a posterior stabilized (PS) TKA and one to a rotating hinge design because of the presence of a ruptured MCL.

**Conclusion:**

Further episodes of dislocation or instability will be prevented by identifying and treating major causes of instability. The increase in the level of constraint and correction of previous technical mistakes is mandatory.

## INTRODUCTION

Although instability remains one of the main reasons for early revision of total knee arthroplasty (TKA), complete dislocation of the knee is a rare but dreaded complication. TKA dislocations have been described with unicompartmental, mobile-bearing, cruciate-retaining (CR), posterior-stabilized (PS), and semiconstrained designs,^2^–^7^ although the designs that retain the posterior cruciate ligament (PCL) are the ones that are most commonly involved with this complication.

The prevalence of knee dislocation following TKA with early PS designs ranged from 1% to 2%, but this has fallen to 0.15–0.5% with newer designs that incorporate changes in the height of the tibial polyethylene post and its anterior translation.^3^,^4^ The most frequently reported causes of instability and TKA dislocation are implant malpositioning, flexion–extension gap mismatch, excessive soft tissue release (usually in valgus deformity, with an exhaustive posterolateral release), extensor mechanism incompetence (i.e., patellectomy), and inappropriate selection of the primary implant. Late rupture of the PCL, rupture of the polyethylene insert,^8^ breakage of the polyethylene post, and neurologic diseases are less common causes.^9^,^10^ We report six cases of TKA dislocation and analyze the patterns of dislocation and the factors related to each of them.

## MATERIALS AND METHODS

Six patients with dislocation of a TKA, five women and one man, were treated at three different institutions from 1998 to 2006. Five of these patients had suffered posterior dislocation and one an anterior dislocation. In all but one of the cases, the index operation was performed at the author’s institution [Table 1]. Before dislocation, patients complained of pain, giving-way episodes, weakness, joint effusions, and difficulty in climbing stairs. Urgent reduction of the dislocation was performed under general anesthesia and, following a period of immobilization and physical therapy, TKA revision was performed as an elective treatment for residual instability in all patients. There were no recurrences after reoperation; however, one patient developed an infection and arthrodesis was performed, two had an active extension lag of 5°, and another could not extend her knee from a sitting position.

**Table 1:** Clinical details of patients

Age (in years)/Sex	Diagnosis	Cause	Type of prosthesis	Type of revision	Associated procedures	ROM
68/F	OA, 20° valgus	Imbalanced gap, exhaustive release, PCL incompetence	CR, Excel (Traider™)	PS (Nex Gen, Zimmer™), increasing the height of the polythene insert	–	0–95°
65/F	OA, 15° valgus	Imbalanced gap, excessive lateral release, wrong polyethylene insert selection	CR, Profix (Smith-Nephew™)	PS (Profix, S and N™), bigger polyethylene insert height, posterior augments	Extensor mechanism imbrication, reattachment and grafting of patellar tendon	5–100°
73/M	OA, varus	Malrotation, extensor mechanism luxation and incompetence, displaced joint line	CR, Profix revision	Semiconstrained, Nex-Gen LCCK (Zimmer™)	Rebuilding posterior and distal condyles, patellar distal reefing	5–110°
71/F	RA, valgus	Extensor mechanism incompetence. Late failure of MCL	PS (IB II, Zimmer™)	Rotating hinge (MRH. Stryker™)	Insall’s imbrication of extensor mechanism	0–110°, 20° lag from sitting position
70/F	OA, varus	Imbalanced gap, PCL incompetence	CR, Excel (Traider™)	PS, Nex-Gen (Zimmer™)	Reconstruction of posterior condyles	Infection Arthrodesis
65/F	OA varus	Imbalanced gap, tibial varus malpositioning	CR, Duraron (Stryker™)	PS (Genesis II, S and N™)	Transient peroneal nerve palsy, ascending geniculate occlusion	0–100°

OA: Osteoarthritis; CR: Cruciate-retaining; PS: Posterior-stabilized; MCL: Medial collateral ligament; PCL: Posterior cruciate ligament; ROM: Range of motion; M: Male, F: Female

### Case 1

A 68-year-old woman with osteoarthritis of knee and a 20° valgus deformity underwent TKA replacement using a CR design. Clinical examination revealed lateral instability in extension and a posterior drawer test greater than 1 cm. No apparent malpositioning of the components was visible on the radiographs. Eight months after the index procedure the knee dislocated and was revised with a PS design. The main presumed cause of the dislocation was flexion–extension gap mismatch due to excessive release of the lateral structures, leaving an incompetent PCL. The use of a CR design in this difficult case could be considered a contributing factor. The knee is stable and pain free 9 years after the revision.

### Case 2

**Figure 1:**
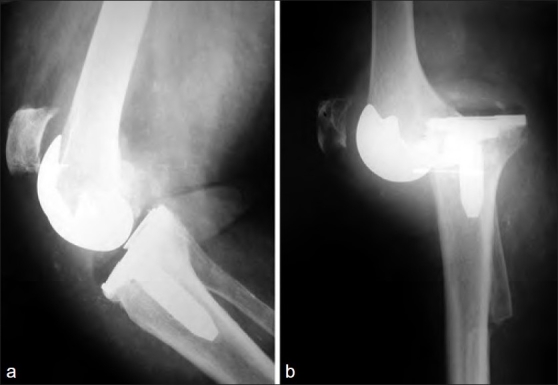
(a) Reduced prostheses after dislocation. Notice the high position of the patella and (b) Dislocation and patellar tendon rupture. Radiological presentation at the emergency room

A 65-year-old woman with osteoarthritis of her right knee and a 15° valgus deformity underwent TKA with a CR design. Radiographs revealed poor offset restitution, with clinical lateral instability both in flexion and in extension. She also had moderate recurvatum and a positive result in the posterior drawer test. Two years after the index operation she had a minor injury and suffered a dislocation of the TKA, with patellar tendon avulsion [Figure 1]. A PS TKA design was chosen; this involved releasing the medial structures, rebuilding the posterior condyles, and using a thicker polyethylene insert. An Achilles tendon allograft was used to reinforce the patellar tendon repair. Excessive lateral release, flexion–extension gap mismatch, and inadequate selection of the polyethylene insert during the index arthroplasty had all contributed to the TKA dislocation. The knee is pain free and stable 11 years after the revision but an extension lag of 5° remains.

### Case 3

A 73-year-old man with left knee osteoarthritis was operated on using a CR design. Ten years later the prosthesis was revised because of aseptic loosening, and an ultracongruent design was used without the use of femoral posterior or distal augments. The radiograph showed an almost laterally dislocated patella, a proximally displaced joint line, a thick polyethylene insert, and poor posterior offset restitution, with a positive posterior drawer test result. The patient suffered a knee dislocation after a minor injury while his leg was in flexion. A semiconstrained design using distal and posterior augments was selected for revision of the TKA. The patellar tendon was reinforced with an Achilles tendon graft. Malrotation of the components and flexion–extension gap mismatch were the main causes for the dislocation, but incorrect restitution of joint line level and extensor mechanism incompetence may have contributed to the dislocation. The knee is stable and pain free 6 years after the revision arthroplasty but a 5° extension lag remains.

### Case 4

A 71-year-old woman who had undergone a patellectomy 20 years earlier developed osteoarthritis with a valgus deformity of the knee joint. TKA was done using a PS design. There was no malpositioning of the components. Five years later the patient experienced significant knee pain and external rotation of the knee with progressive failures, leading to rupture of the medial collateral ligament (MCL). The patient was unable to extend the knee completely. The knee dislocated after a giving-way episode. A rotating hinge prosthesis design was used during the revision procedure and the extensor mechanism was reinforced with Insall’s proximal imbrication. Six years after the revision arthroplasty the patient has an extension lag of 20° and cannot stabilize the rotating knee mechanism of the prostheses to optimize traction force. Mediolateral instability, with failure of the MCL and extensor mechanism incompetence were the main causes for the dislocation.

### Case 5

A 70-year-old woman with varus osteoarthritis of her left knee was operated on using a CR design. Clinical examination revealed medial instability in flexion, with a positive posterior drawer test result. Radiographs revealed no malpositioning of components. Flexion-extension gap mismatch, incompetent PCL and inadequate posterior off-set restitution were considered the contributing factors. She suffered a posterior dislocation 6 months after the index operation and the knee was revised with a PS TKA design. She developed an early infection treated with two early debridement procedures and polyethylene exchange without success. A two-stage revision was performed but the knee dislocated with two types of cement-loaded spacers. The extensor mechanism and collateral ligaments were damaged and the knee was finally arthrodesed. The follow up is 4 years. Inadequate flexion–extension balance and poor offset restitution were considered the reasons for dislocation.

### Case 6

**Figure 2a-c:**
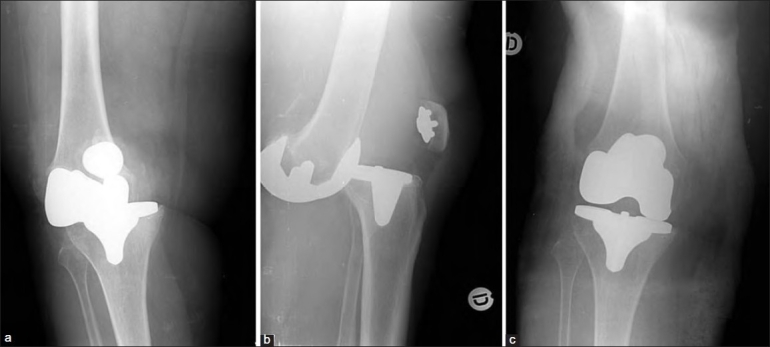
Case 6. Anterior dislocation. Malposition of the tibial component and flexion–extension imbalance. Notice the patellar tendon has not been avulsed, the patella remains at its theoretical position

A 65-year-old woman with varus osteoarthritis of the right knee was operated on using a CR design. Examination revealed a varus deformity with recurvatum and flexion instability. X-rays showed a varus positioning of the tibial tray greater than 5°. She later suffered anterior knee dislocation, with occlusion of the ascending geniculate artery and peroneal palsy. The knee was revised using a PS design with a thicker polyethylene without associated procedures. The knee is stable and pain free 6 years after the revision arthroplasty. Malpositioning of the tibial component, excessive internal rotation and varus angulation of the tibial component, and flexion–extension imbalance were considered the factors contributing to TKA dislocation [Figure 2a–c].

## DISCUSSION

Clinical instability has been estimated to be present in 1%–2% of patients following a TKA procedure and in 10%–20% after a TKA revision. TKA dislocation, although rare, is the worst possible form of instability.^1^

The leading cause for instability after TKA was considered on the bases of clinical examination rather than on X-rays findings; Table 2 summarize the factors and the patterns of instability. We have only considered grossly incorrect malpositioning (i.e., tibial tray in varus) greater than 5° or subluxated patella in one patient with malrotated component and check preoperative X-rays with intraoperative findings. We agree that minor malpositioning is difficult to determine and its clinical relevance is often uncertain. The authors have attributed these causes according to their clinical experience and the radiologic findings.

**Table 2:** Patterns of instability and contributing factors

Mediolateral instability
Malpositioning
Ligament imbalance
Inadequate implant selection
Anteroposterior instability (Rare in extension, usually associated with flexion instability)
Traumatism
Polyethylene post breakage
Hyperextension
Extensor mechanism incompetence
Flexion instability
Early form (Usually associated with PCL incompetence, AP and ML instability)
Flexion–extension mismatch
Poor offset restitution: Small femoral component, anterior or extension placement
Excessive tibial posterior slope
Thin polyethylene insert to compensate for thigh extension gap
Displacement of the joint line making the collateral ligaments non functional
Iatrogenic damage of PCL or exhaustive release of posterolateral structures
Inadequate implant selection
Late form (AP, not ML flexion instability)
Late rupture or degeneration of the PCL
Extensor mechanism incompetence
Rotational instability
Rotational instability (Usually associated with flexion instability)
Implant malpositioning
Collateral ligament imbalance

### A. Mediolateral instability

Although mediolateral (ML) or coronal plane instability may be as frequent a reason for revision of TKA as anteroposterior (AP) instability, it is only rarely the sole cause for a TKA dislocation.^2^,^11^ ML instability can be due to iatrogenic injury, incorrect ligament balancing,^11^ or lack of identification of an incompetent collateral ligament. In an otherwise well-positioned implant, late ML instability is usually related to implant loosening or malalignment leading to ligament incompetence, rather than to soft tissue imbalance.^12^

### B. Anteroposterior instability

AP instability in extension is rare, even when there is marked soft tissue laxity, because of the axial loads and the conformity of the prosthetic components. Extensor mechanism incompetence, inadequate balancing of the PCL, excessive release of posterolateral structures, polyethylene post rupture, hyperextension, a broken polyethylene insert anterior to the post, or a direct traumatism may all contribute to anteroposterior TKA dislocation.^1^,^8^

### C. Flexion instability

Early flexion instability usually affects both the AP and ML planes, and the most common causes are flexion–extension gap mismatch, iatrogenic damage of the PCL, or both.

**Figure 3:**
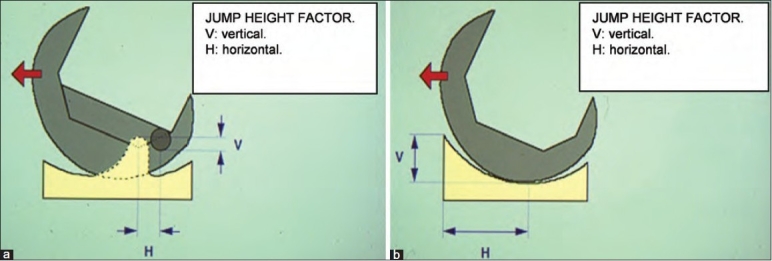
A line diagram showing (a) ‘Jump height factor’ for a PS and (b) for a deep dish component. Notice the jump height factor is greater for an ultracongruent design than for a PS one

In the setting of a flexion–extension gap mismatch, two mechanisms for dislocation have been proposed: 1) A rotary dislocation may occur in patients with a valgus deformity when an exhaustive lateral release has injured the popliteus tendon and the lateral collateral ligament; and 2) a strong contraction of the hamstring while the knee is in flexion can cause a jump of the femoral component over the tibial polyethylene insert [Figure 3].^5^,^13^

Four of the six patients presented in this series (cases 1, 2, 5, 6), all with CR implants, had flexion–extension gap mismatch. Contributing causes included exhaustive surgical release, poor posterior offset restitution, PCL incompetence, or component malpositioning.

Late forms of flexion instability may be associated with delayed rupture or degeneration of the PCL, extensor mechanism incompetence, and rotational instability. Late PCL incompetence causes instability that is usually restricted to the AP plane. In association with posterior capsule incompetence and secondary knee recurvatum, a deformity may cause TKA dislocation.^14^

An extensor mechanism failure may contribute to TKA instability and posterior dislocation.^2^ Our fourth case had a previous patellectomy before implantation of a PS TKA; she developed progressive attenuation of both the quadriceps and the MCL until the latter finally ruptured and the TKA was dislocated. The extensor mechanism was imbricated during the revision arthroplasty, but the patient was not able to fully extend her knee actively even though the design used (MRH, Stryker™) had a posteriorly placed hinge that is supposed to increase the lever arm of the extensor mechanism. Reconstructive techniques for the extensor mechanism are commonly necessary after TKA dislocation. A distal reinforcement of the patellar tendon was performed in our second and third cases.

### D. Rotational instability

Rotational instability due to ligament imbalance or component malpositioning can also lead to dislocation. An abnormal external rotation of the femoral component or internal rotation of the tibial component causes rotational instability in flexion of greater than 45°.^15^,^16^

Two out of our six patients had malpositioning of one component (varus and internal rotation of tibial tray ≥ 5°, in case 6) or both components (severe internal rotation of both components with an almost dislocated patella, poor posterior offset restitution, and a proximal joint line, as in case 3).

## TREATMENT OF DISLOCATION

Adequate identification of the causes leading to TKA dislocation is mandatory for successful treatment. As a general rule, at knee revision arthroplasty, the surgeon must increase at least one grade the constriction of the implant design.

**Figure 4a-b:**
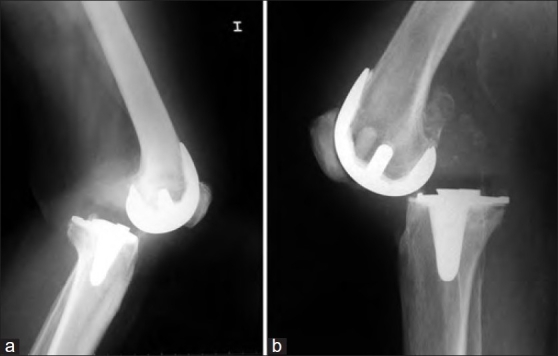
X-ray (lateral view) of knee joint showing posterior dislocation. Patellar tendon has not been avulsed. Imbalanced gap with PCL incompetence were the main contributing factors. Cases 1 and 5

In the presence of a CR design [Figure 4a and b], simply increasing the height of the polyethylene insert or replacing it with an ultracongruent polyethylene insert has been associated with a high percentage of failures (30%–35% at 5 years).^17^,^18^ Complete exchange and implantation of a PS design is the ‘gold standard.’ Pagnano *et al*. treated 22 patients with this approach and reported an 86% success rate.^19^,^20^

In the setting of flexion instability with a PS design, immobilization and muscular strengthening may be sufficient to provide adequate knee stability. Lombardi *et al*.,^3^ in a series of 15 patients with these characteristics, reported 11 good results. Diduch^21^ obtained similar results in two out of three patients. Increasing the height of the polyethylene insert occasionally solves the problem, but in the presence of severe laxity, flexion–extension mismatch, or component malpositioning, a complete revision or an intercondylar constrained design is necessary.^22^

We tried to use the lesser degree of constrain as possible, but this usually requires to increase the constrain by one degree. However, in two of our patients we needed to increase two levels (cases 3 and 4). The decision regarding the degree of constriction chosen for revision was determined after taking into account the causative factors of the dislocation. The authors used the brand authorized at our hospitals (blinded and unrestricted public offers) for the intended purpose.

Five out of six patients in our series had a CR TKA designs: four were revised to a PS TKA and one to a rotating hinge design because of the presence of a ruptured MCL. One case with an ultracongruent design was revised with a condylar constrained design, including the use of distal and posterior augments.

The use of a more constrained TKA design *per se* is not enough to prevent dislocation when there are persistent associated causes that must be addressed or when other conditions such as neurologic disorders are present.^5^,^6^
